# Recommendations for the diagnosis and treatment of paroxysmal kinesigenic dyskinesia: an expert consensus in China

**DOI:** 10.1186/s40035-021-00231-8

**Published:** 2021-02-16

**Authors:** Li Cao, Xiaojun Huang, Ning Wang, Zhiying Wu, Cheng Zhang, Weihong Gu, Shuyan Cong, Jianhua Ma, Ling Wei, Yanchun Deng, Qi Fang, Qi Niu, Jin Wang, Zhaoxia Wang, You Yin, Jinyong Tian, Shufen Tian, Hongyan Bi, Hong Jiang, Xiaorong Liu, Yang Lü, Meizhen Sun, Jianjun Wu, Erhe Xu, Tao Chen, Tao Chen, Xu Chen, Wei Li, Shujian Li, Qinghua Li, Xiaonan Song, Ying Tang, Ping Yang, Yun Yang, Min Zhang, Xiong Zhang, Yuhu Zhang, Ruxu Zhang, Yi Ouyang, Jintai Yu, Quanzhong Hu, Qing Ke, Yuanrong Yao, Zhe Zhao, Xiuhe Zhao, Guohua Zhao, Furu Liang, Nan Cheng, Jianhong Han, Rong Peng, Shengdi Chen, Beisha Tang

**Affiliations:** 1 Department of Neurology, Ruijin Hospital, Shanghai Jiao Tong University School of Medicine, Shanghai, China; 2 Department of Neurology, The Sixth People’s Hospital, Shanghai Jiaotong University, Shanghai, China; 3 Department of Neurology and Institute of Neurology, The First Affiliated Hospital, Fujian Medical University; Fujian Key Laboratory of Molecular Neurology, Fujian Medical University, Fuzhou, China; 4 Department of Neurology and Research Center of Neurology, The Second Affiliated Hospital; Key Laboratory of Medical Neurobiology of Zhejiang Province, Zhejiang University School of Medicine, Hangzhou, China; 5 Department of Neurology, The First Affiliated Hospital, Sun Yat-sen University, Guangdong Provincial Key Laboratory of Diagnosis and Treatment of Major Neurological Diseases; National Key Clinical Department and Key Discipline of Neurology, Guangzhou, China; 6 Department of Neurology, China-Japan Friendship Hospital, Movement Disorder and Neurogenetics Research Center, Beijing, China; 7 Department of Neurology, Shengjing Hospital of China Medical University, Shenyang, China; 8 Department of Neurology, The First Affiliated Hospital of Xinjiang Medical University, Urumqi, China; 9 Department of Neurology, The First Affiliated Hospital of Anhui Medical University, Collaborative Innovation Center for Neuropsychiatric Disorders and Mental Health, Anhui Medical University; Anhui Province Key Laboratory of Cognition and Neuropsychiatric Disorders, Hefei, China; 10 Department of Neurology, Xijing Hospital, Fourth Military Medical University, Xi’an, China; 11 Department of Neurology, The First Affiliated Hospital of Soochow University, Suzhou, China; 12 Department of Neurology, The First Affiliated Hospital of Nanjing Medical University, Nanjing, China; 13 Department of Neurology, The First Affiliated Hospital of Guangxi Medical University, Nanning, China; 14 Department of Neurology, Peking University First Hospital, Beijing, China; 15 Department of Neurology, Changzheng Hospital, the PLA Naval Medical University, Shanghai, China; 16 Department of Neurology and Department of Emergency, Guizhou Provincial People’s Hospital, Guiyang, China; 17 Department of Neurology, Affiliated Hospital of Guizhou Medical University, Guiyang, China; 18 Department of Neurology, Beijing Friendship Hospital, Capital Medical University, Beijing, China; 19 Department of Neurology, Xiangya Hospital, Central South University, Changsha, China; 20 National Clinical Research Center for Geriatric Diseases, Central South University; Key Laboratory of Hunan Province in Neurodegenerative Disorders, Central South University; Laboratory of Medical Genetics, Central South University, Changsha, China; 21 Institute of Neuroscience and The Second Affiliated Hospital of Guangzhou Medical University, Key Laboratory of Neurogenetics and Channelopathies of Guangdong Province and the Ministry of Education of China, Guangzhou, China; 22 Department of Geriatrics, The First Affiliated Hospital of Chongqing Medical University, Chongqing, China; 23 Department of Neurology, the First Hospital of Shanxi Medical University, Taiyuan, China; 24 Department of Neurology, Huashan Hospital, Fudan University, Shanghai, China; 25 National Clinical Research Center for Aging and Medicine, Huashan Hospital, Fudan University, Shanghai, China; 26 Department of Neurology, Xuanwu Hospital, Capital Medical University, National Clinical Research Center for Geriatric Diseases, Beijing, China; 27 Department of Neurology, Hainan General Hospital, Haikou, China; 28 Department of Neurology, First Affiliated Hospital of Kunming Medical University, Kunming, China; 29 Department of Neurology, Shanghai Eighth People’s Hospital Affiliated to Jiang Su University, Shanghai, China; 30 Department of Neurology, Beijing Tiantan Hospital, Capital Medical University; China National Clinical Research Center for Neurological Diseases; Center of Stroke, Beijing Institute for Brain Disorders; Beijing Key Laboratory of Translational Medicine for Cerebrovascular Disease, Beijing, China; 31 Department of Neurology, Henan Provincial People’s Hospital, Zhengzhou University People’s Hospital, Zhengzhou, China; 32 Department of Neurology, Affiliated Hospital of Guilin Medical University, Guilin Medical University; Guangxi Key Laboratory of Brain and Cognitive Neuroscience, Guilin Medical University, Guangxi Clinical Research Center for Neurological Diseases, Affiliated Hospital of Guilin Medical University, Guilin Medical University; Guangxi Key Laboratory of Brain and Cognitive Neuroscience, Guilin Medical University, Guilin, China; 33 Department of Neurology and Neuroscience Center, First Hospital of Jilin University, Changchun, China; 34 Department of Neurology, The First Affiliated Hospital of Harbin Medical University, Harbin, China; 35 Department of Neurology, and Neuroscience Center, General Hospital of Ningxia Medical University, Key Laboratory of Craniocerebral Diseases of Ningxia Hui Autonomous Region, Yinchuan, China; 36 Department of Neurology, Jiangxi Provincial People’s Hospital Affiliated with Nanchang University, Nanchang, China; 37 Department of Neurology, Tongji Hospital, Tongji Medical College, Huazhong University of Science and Technology, Wuhan, China; 38 Department of Geriatrics and Neurology, The Second Affiliated Hospital and Yuying Children’s Hospital, Wenzhou Medical University, Wenzhou, China; 39 Department of Neurology, Guangdong Neuroscience Institute, Guangdong Provincial People’s Hospital, Guangdong Academy of Medical Sciences, Guangzhou, China; 40 Department of Neurology, The Third Xiangya Hospital, Central South University, Changsha, China; 41 Department of Neurology, First Affiliated Hospital, China Medical University, Shenyang, China; 42 Department of Neurology, Qinghai Provincial People’s Hospital, Qinghai, China; 43 Department of Neurology, The First Affiliated Hospital, Zhejiang University School of Medicine, Hangzhou, China; 44 Department of Neurology, Guizhou Provincial People’s Hospital, Medical School of Guizhou University, Guiyang, China; 45 Department of Neurology, the Third Hospital of Hebei Medical University, Shijiazhuang, China; 46 Department of Neurology, Qilu Hospital, Shandong University, Jinan, China; 47 Department of Neurology, the Fourth Affiliated Hospital, Zhejiang University School of Medicine, Yiwu, China; 48 Department of Neurology, Baotou Central Hospital, Baotou, China; 49 Institute of Neurology, Hospital Affiliated with Anhui University of TCM, Hefei, China; 50 Department of Neurology, Second Affiliated Hospital of Kunming Medical University, Kunming, China; 51 Department of Neurology, West China Hospital, Sichuan University, Chengdu, China; 52 State Key Laboratory of Medical Genetics, Changsha, China

**Keywords:** Paroxysmal kinesigenic dyskinesia, Diagnosis and treatment, Expert consensus, China

## Abstract

Paroxysmal dyskinesias are a group of neurological diseases characterized by intermittent episodes of involuntary movements with different causes. Paroxysmal kinesigenic dyskinesia (PKD) is the most common type of paroxysmal dyskinesia and can be divided into primary and secondary types based on the etiology. Clinically, PKD is characterized by recurrent and transient attacks of involuntary movements precipitated by a sudden voluntary action. The major cause of primary PKD is genetic abnormalities, and the inheritance pattern of PKD is mainly autosomal-dominant with incomplete penetrance. The proline-rich transmembrane protein 2 (*PRRT2*) was the first identified causative gene of PKD, accounting for the majority of PKD cases worldwide. An increasing number of studies has revealed the clinical and genetic characteristics, as well as the underlying mechanisms of PKD. By seeking the views of domestic experts, we propose an expert consensus regarding the diagnosis and treatment of PKD to help establish standardized clinical evaluation and therapies for PKD. In this consensus, we review the clinical manifestations, etiology, clinical diagnostic criteria and therapeutic recommendations for PKD, and results of genetic analyses in PKD patients performed in domestic hospitals.

## Introduction

Paroxysmal dyskinesias are a group of neurological diseases characterized by intermittent episodes of involuntary movements with different causes. The involuntary movements are manifested as dystonia, chorea, ballism, or a combination thereof. According to the triggers of the attack, paroxysmal dyskinesia can be divided into types of paroxysmal kinesigenic dyskinesia (PKD), paroxysmal nonkinesigenic dyskinesia (PNKD), and paroxysmal exercise-induced dyskinesia (PED). PKD is the most common type of paroxysmal dyskinesia and was first described by Kertesz in 1976 [1]. Clinically, PKD is characterized by recurrent and transient episodes of involuntary movements precipitated by a sudden voluntary action [1]. PKD can be divided into primary and secondary types based on the etiology. The primary PKD is mainly an inherited condition, with most cases having an inheritance pattern of autosomal-dominant with incomplete penetrance [2, 3]. Proline-rich transmembrane protein 2 (*PRRT2*) was the first identified causative gene in 2011, accounting for the majority of PKD patients worldwide [4–6]. Subsequent studies have revealed clinical and genetic characteristics of PKD. To help standardize PKD clinical evaluation and therapies, we considered the views of domestic experts and propose an expert consensus regarding the diagnosis and treatment of PKD.

## Clinical manifestations

### Prevalence and age of onset

The prevalence of PKD has been estimated to be 1:150,000 individuals. The majority of PKD individuals are of Asian ethnicity and are from China and Japan, followed by North America and Europe [7, 8]. The age at onset of primary PKD generally ranges from several months to 20 years, with a particularly high incidence among 7- to 15-year-old children and adolescents [2, 8, 9]. Males are more susceptible to primary PKD than females, with a ratio of 2:1 to 4:1 [2]. A higher prevalence of sporadic PKD has been reported in males, with a ratio from 4:1 to 8:1 [10].

### Triggers

Typical PKD attacks are induced by sudden voluntary actions, such as sudden standing, starting to run, getting on and off a car, and encountering traffic lights [2, 9, 10]. Changes in the speed or amplitude of movements, addition of another type of movement during an activity, or even the intent to move can also cause an attack. Episodes are more likely to be triggered when an individual is under emotional stress, stimulated by a sound or image, or hyperventilating [10].

### Aura

The aura of PKD is defined as abnormal sensations prior to the appearance of involuntary movements induced by a sudden movement or movement intention [9]. The abnormal sensation is a feeling that is difficult to describe accurately and differs by individual. Approximately 78–82% of patients with PKD may experience aura [9]. The most common descriptions of aura are numbness, tingling, and muscle weakness [2, 9]. Some patients have been able to alleviate the dyskinesia attack by slowing their movements when experiencing aura [10]. In some cases, aura appears in isolation without subsequent dyskinesia attacks [9].

### Attack forms

The forms of a PKD attack include unilateral or bilateral dystonia, chorea, ballism or a mixture of them [2, 10]. Dystonia is the most common, followed by chorea and ballism, which is the rarest form of PKD [9, 11]. Face involvement has been reported by approximately 70% of patients, mainly manifesting as face twitching, rigidity of facial muscles and dysarthria, which may be related to the dystonia of facial or laryngeal muscles [9, 12].

### Duration and frequency of attacks

The duration of PKD attacks is < 1 min in over 98% of patients [2, 9]. For patients with prolonged duration, the secondary factors of PKD should be excluded [9]. The frequency of PKD attacks varies significantly among individuals and in patients with different disease stages, ranging from several times a year to more than 100 times per day [9, 12, 13]. The frequency of PKD attacks usually peaks during puberty and decreases after the age of 20 years. Some patients rarely experience attacks or even experience spontaneous remission of the disease after the age of 30 years [9].

### Clinical classification

Clinically, PKD is classified into the pure and complicated types according to the absence or presence of other symptoms or diseases.

Patients of the pure form present only with kinesigenic involuntary movements.

Patients with complicated type of PKD present with neurological symptoms in addition to the kinesigenic dyskinesia. These combined manifestations include benign familial infantile epilepsy (BFIE), febrile convulsion, migraine, hemiplegic migraine, episodic ataxia, epilepsy and other episodic diseases [2, 14–16]. A few patients exhibit developmental delay, intellectual deficit, language dysfunction or autism [17–19].

### Etiology and pathogenesis

PKD can be classified into the primary and secondary PKD due to different causes [20]. The primary PKD is further categorized into familial and sporadic PKD. Currently, the primary familial PKD is considered to be primarily caused by genetic factors with autosomal dominant inheritance, accompanied by incomplete penetrance that is estimated to be 60–90% [21]. Genetic studies have identified multiple genes related to the pathogenesis of PKD, including *PRRT2, PNKD, SLC2A1, SCN8A, KCNMA1, KCNA19* and *DEPDC5* [4, 5, 15, 22–28].

*PRRT2* is the major causative gene for PKD [8, 9, 15]. It is located on chromosome 16p11.2 and contains 4 exons, encoding 340 amino acids. To date, more than 80 mutations of *PRRT2* have been reported worldwide [9], with nonsense and frameshift mutations being the main types, followed by missense mutations. Among the documented mutations, c.649dupC is a hotspot [9, 12, 29]. However, the function and pathogenic mechanism of PRRT2 remain unclear. PRRT2 is an integral component of the SNARE complex, interacting with SNAP-25, synaptic binding protein-1, synaptic binding protein 2 and synaptic vesicle protein 2, which endows the SNARE complex with calcium sensitivity [30, 31]. Furthermore, PRRT2 is a key negative modulator of Nav1.2 and Nav1.6 channels [32]. The abnormal basal ganglia−thalamic−cortical circuit is currently considered to be the pathophysiological basis of PKD [33–36]. Functional magnetic resonance imaging (MRI) studies have revealed an abnormal connectivity between the thalamus and the motor cortex in patients with PKD, and the functional abnormality is associated with the duration of the disease [37]. In patients with *PRRT2* mutations, the thalamo−prefrontal hypoconnectivity has been observed, indicating that the *PRRT2* mutations result in inefficient thalamo-prefrontal integration and dysfunction of motor inhibition [38]. Mechanistic studies have also revealed that the core pathogenesis of PKD is the disturbed cell excitability caused by *PRRT2* mutation, which is associated with presynaptic dysfunction, abnormal neurotransmitter release and the lack of negative regulation of Na^+^ channels [31, 32].

Still, approximately one-half of patients with primary PKD do not harbor mutations in the aforementioned genes, suggesting the existence of other disease-causing genes.

In a few cases, PKD may be secondary to other factors [39], such as demyelinating diseases of the central nervous system, cerebrovascular diseases, traumatic brain injury, or metabolic abnormalities [39–41]. Multiple sclerosis (MS), particularly the relapsing-remitting MS, is the most common cause of secondary PKD [42–46]. The lesions of MS related to PKD involve the thalamus, the lenticular nucleus, the globus pallidus and the internal capsule [43], and these demyelinating lesions may result in increased axon sensitivity that causes symptoms [43]. Calcification of the basal ganglia, including the idiopathic basal ganglial calcification and the basal ganglial calcification secondary to hypoparathyroidism or pseudo-parathyroidism, may also cause the secondary PKD [47–51].

## Diagnosis and differential diagnosis

### Differential diagnosis

#### a. Epilepsy

Although PKD attacks are stereotypic, precipitated by certain factors and not accompanied by loss of consciousness, it is difficult to distinguish them from seizure disorders, particularly the frontal lobe epilepsy. Patients with seizure disorders present with an abnormal ictal or interictal electroencephalogram (EEG) or no EEG change. The frontal lobe epilepsy is a common type of focal epilepsy of the childhood. Some patients with frontal lobe epilepsy also present with recurrent and stereotypic chorea and dystonia, with slight disturbance of consciousness during the attack and sometimes normal interictal electrograms. Unlike the frontal lobe epilepsy, however, the PKD attacks have a clear kinesigenic trigger and the individuals remain conscious during the attack, which can be used to distinguish between the two disorders. In addition, seizures of the frontal lobe can occur both during wakefulness and more commonly in sleep, while PKD is only evident when patients are awake.

#### b. Primary PNKD

The onset of PNKD usually occurs in childhood, with clinical features of involuntary movements triggered by nonkinesigenic factors such as tea, coffee, alcohol, psychological stress and fatigue. The attack presents with unilateral or bilateral dystonia and chorea. PNKD attacks usually last longer than PKD attacks, ranging from 10 min to 1 h [2, 52]. A few patients may experience even longer attacks. The frequency of PNKD attacks is lower than that of PKD attacks. Approximately one-half of the patients may experience aura prior to a PNKD attack, similar to that in PKD.

#### c. PED

The age at onset of primary PED is between 2 and 30 years. PED attack is induced by long or continuous exercise (5–30 min), but not by nonkinesigenic factors such as cold, alcohol, or coffee [53]. The duration of the attack ranges from 5 min to 45 min, typically not exceeding 2 h.

#### d. Psychological movement disorders and pseudoseizures

Both psychological disorders and PKD can manifest as paroxysms with normal interictal neurological examinations. Because of the clinical characteristics of PKD, attacks are usually not witnessed by physicians. Moreover, most patients with PKD are also diagnosed with anxiety or depression [54]. Therefore, in some cases, it is difficult to distinguish psychogenic movement disorders and pseudoseizures from PKD. Psychogenic disorders have features of distractibility, variability of clinical presentations of different paroxysms, and suggestibility [55]. Other red flags for suspecting psychogenic disorder include adult age of onset, altered level of responsiveness during attacks, additional psychogenic physical signs, medically unexplained somatic symptoms, and an atypical response to medications [55, 56]. Administering a high-knee exercise test may also help physicians make differential diagnoses.

#### e. Tics

Tics are very brief jerks or dystonic postures that are typically shorter in duration than PKD attacks.

#### f. Hyperekplexia

Hyperekplexia manifests as a group of diverse, complex, abnormal movements triggered by sudden noise or touch that can mimic PKD [55, 57]. An excessive startle response (typically including eye blinking and a flexor spasm of the trunk) to unexpected and innocuous (particularly auditory) stimuli is the most striking feature of hyperekplexia, which is present from birth or evident prenatally in the last trimester [58]. In contrast to the physiological startle response, the excessive startle leads to prolonged stiffening in neonates and young infants [59, 60].

#### g. Sandifer syndrome

Sandifer syndrome is secondary to gastroesophageal reflux, and the diagnosis of Sandifer syndrome should be suspected in young children with paroxysms of head tilt after eating [55, 61].

#### h. Benign paroxysmal torticollis (BPT) [62]

BPT is present as recurrent episodes of abnormal, painless head postures, alternating from side to side. Attacks may last from a few minutes to several days. Onset usually occurs before 3 months of age, and migraines that appear later have been widely reported, suggesting that BPT should be considered as an age-dependent migraine disorder to include periodic syndromes of childhood [63]. Treatment is not usually needed unless the symptoms of irritability, discomfort, or vomiting necessitate symptomatic management.

#### i. Transient dystonia of infancy [62]

Transient dystonia of infancy consists of paroxysmal episodes of abnormal upper limb posture, with occasional concomitant involvement of the trunk and a single lower limb [64]. The interictal examination and neuroimages are normal. The age of onset is typically between 5 and 10 months, and the condition gradually resolves between 3 months and 5 years without developmental or neurological abnormalities. The etiology and pathophysiology of transient dystonia of infancy are unclear.

#### j. Benign myoclonus of early infancy (BMEI) [62]

BMEI was originally described as a nonepileptic paroxysmal motor disorder characterized by the occurrence of myoclonic jerks of the head and/or of the upper limbs, usually occurring in clusters and mimicking infantile spasms. Consciousness is preserved during attacks, which usually occur during wakefulness and more rarely during sleep or drowsiness. Ictal EEG, neurological status and development must be normal to confirm a BMEI diagnosis. The attacks usually have abrupt onset and frequently appear in clusters. Each attack usually lasts a few seconds, but multiple episodes per day often occur and are frequently triggered by excitement, frustration, postural changes, or sensory stimuli [63]. The onset occurs in the first year of life (mainly between 4 and 7 months), and attacks usually cease by the age of 2 years, although sometimes persisting into the childhood. The pathophysiology is unknown, and no treatment is needed, but parents may need reassurance.

## Clinical diagnostic criteria

Based on the results of a large-scale clinical study of patients with PKD in China [9], we propose the modified clinical diagnostic criteria for PKD (Table 1), detailed as below:

1. Patients suspected of having PKD must present with all core symptoms.
2. Supportive evidence endorses the diagnosis of PKD.
3. Secondary factors such as vascular, demyelinating, and metabolic causes must be excluded. In the case of appearance of any red flags, a comprehensive evaluation should be conducted to exclude secondary PKD.

**Table 1:** Revised clinical diagnostic criteria for primary PKD

**Core symptoms:**	
1. Kinesigenic triggers and attacks presenting as dystonia, chorea, ballism, or a combination of them; 2. No impairment of awareness during attacks.	
**Supportive evidence:**	
1. Presence of aura; 2. Attack duration < 1 min; 3. Positive result of high-knee exercise test; 4. Good response to low-dose voltage-gated sodium channel blockers, especially carbamazepine/ oxcarbazepine.	
**Diseases listed in the following should be excluded:** 1. Cerebrovascular disease; 2. Demyelinating disease, especially multiple sclerosis; 3. Metabolism disorders: a. Hyperthyroidism; b. Calcium-phosphate metabolism disorders (hypoparathyroidism, pseudoparathyroidism, parathyroid hyperthyroidism, pseudoparathyroid hyperplasia), primary familial brain calcification; c. Glucose metabolism disorder; d. Kernicterus 4. Brain trauma; 5. Psychological disorder. **The following red flags may indicate secondary causes or an alternative diagnosis:** 1. Duration of attacks > 1 min; 2. Age of onset over 20 years; 3. Abnormalities in brain CT/MRI scanning or the presence of other neurologic/systemic problems; 4. No response to anticonvulsants; 5. Abnormal results of interictal examinations.	

Specific assessments include:

- Thyroid function evaluation: measurement of serum T3/FT3, T4/FT4, and thyroid stimulating hormone (TSH) levels; thyroid ultrasound; and examination of the iodine uptake rate if necessary;
- Assessment of calcium and phosphorus metabolism: measurements of serum calcium, phosphorus, parathyroid hormone and calcitonin levels, and a cerebral CT scan is recommended for assessing intracranial calcification;
- Blood glucose test;
- Test of bilirubin levels;
- Test of serum ceruloplasmin levels;
- Head MRI;
- EEG;
- Neuropsychological assessment.

4) High-knee exercise test [9]: Similar to other epileptic disorders, PKD attacks are seldom witnessed by physicians, and the clinical diagnosis is made mainly based on patients’ statements. An imprecise description of clinical features may lead to a misdiagnosis, and witnessing the attack may significantly improve the accuracy of diagnosis. Therefore, it is recommended that PKD attacks be induced through a high-knee exercise test.
  The operation of the high-knee exercise test [9] requires patients to perform high-knee exercise in place, under observations by neurologists. When performing the high-knee exercise, the patient should immediately stop the exercise upon experiencing an aura, and physicians then record the details of the attack, including the form and the duration of the attack and the presence or absence of facial involvement. If a dyskinesia attack or an aura is induced, the test result is presumed to be positive. If no attack is induced after 30 s of high-knee exercise, the test is terminated and the result is presumed to be negative.
  Notably, if the patient is taking anticonvulsants or his/her symptoms are naturally in remission, attacks may not be induced. Considering the refractory period between two attacks, the results of the high-knee exercise test may also be negative when the exercise is conducted shortly after a previous attack.

### Genetic diagnosis

The primary PKD is mainly attributed to hereditary factors, and the most commonly mutated gene is *PRRT2*. About one-third of patients with primary PKD carry *PRRT2* mutations [9, 12], among which the c.649dupC has received much attention [9, 12, 29] and accounts for 76% of PKD patients with *PRRT2* mutations [9]. Other genes related to episodic diseases can also contribute to PKD, including *PNKD, SLC2A1, SCN8A, KCNMA1, KCNA1,* and *DEPDC5* [4, 5, 15, 22–28].

Since PKD is a benign disease with natural remission and anticonvulsants are effective in controlling attacks, genetic screening is not mandatory and is recommended depending on the patients’ willingness. Second, the diagnosis of PKD is mainly based on clinical features rather than laboratory examinations; thus, negative genetic findings cannot exclude a diagnosis of PKD. Last, genetic screening is not recommended in asymptomatic populations, infants, or unborn babies.

**Fig. 1:**
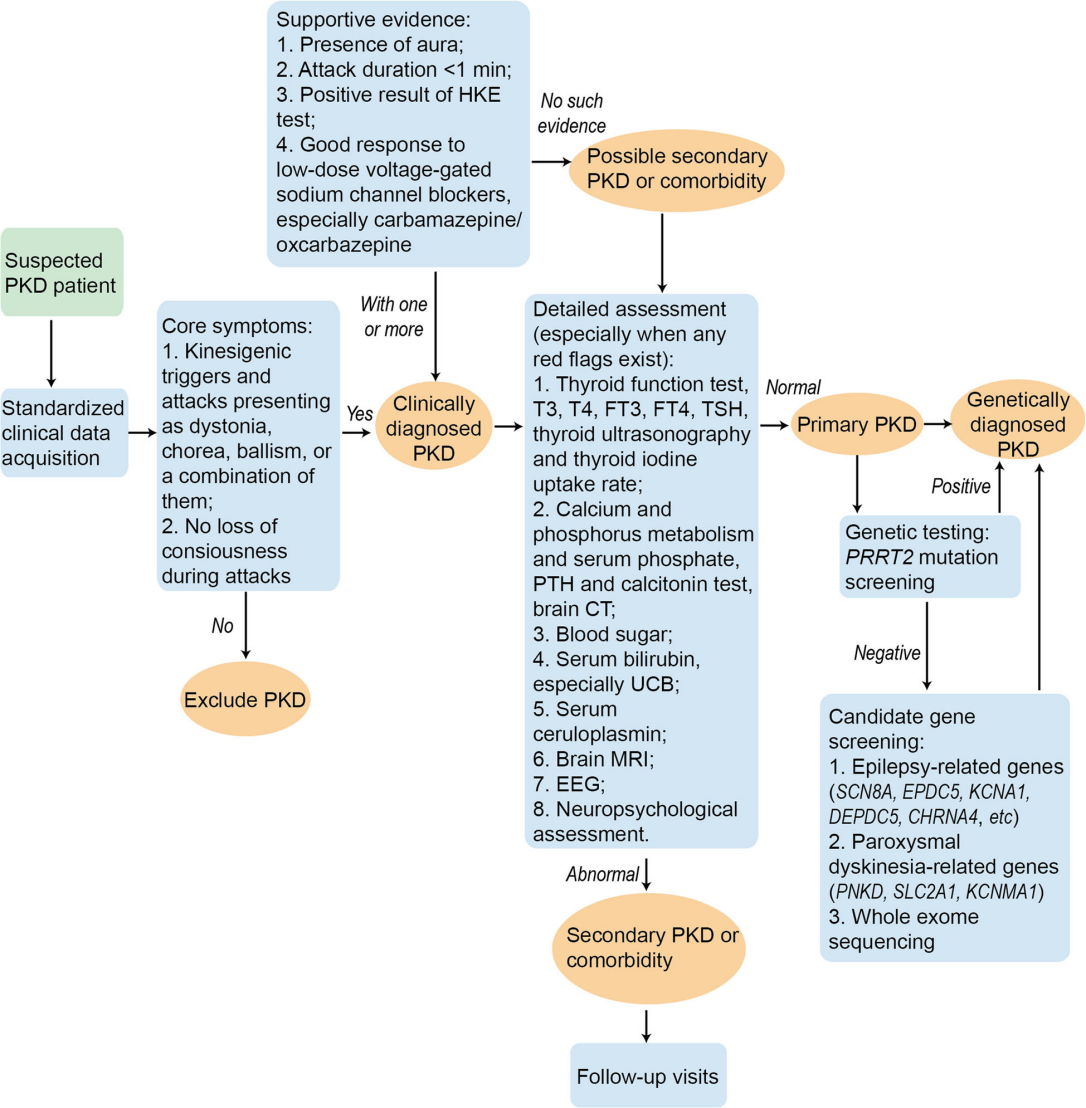
Flowchart for a recommended approach for PKD diagnosis. CT: computed tomography; EEG: electroencephalogram; HKE, high-knee exercise test; MRI: magnetic resonance imaging; PKD: paroxysmal kinesigenic dyskinesia; UCB: unconjugated bilirubin

The recommended flowchart for the diagnosis of PKD is shown in Fig. 1.

## Treatment

The treatment of PKD includes medication and psychotherapy. For patients with secondary PKD, etiological treatment is crucial.

Since the primary PKD is a benign disease with natural remission, the application of medication should be considered on the basis of the patient’s age, the frequency and degree of attacks, the psychological impact of the attack on the patient’s life, and the patient’s willingness.

### Medication

Drugs can be prescribed to patients with frequent and severe attacks (causing instability or even falls), severe psychological impacts, and individuals who are willing to control the attacks. Primary PKD has a dramatic response to anticonvulsants, particularly the sodium channel blockers, of which carbamazepine/oxcarbazepine are preferred [9, 12, 65, 66]. Approximately 97% of patients receiving carbamazepine/oxcarbazepine treatment have reported a complete or partial relief of attacks [9]. More than 85% of patients can achieve complete remission with low-dose carbamazepine (50–200 mg/day) or oxcarbazepine (75–300 mg/day), and approximately 10% of patients can achieve partial control (frequency reduced by at least 75%) [9]. Importantly, the dosage should be flexible, as the extent of satisfaction with the treatment is subjective and individualized. Some patients may tolerate auras without attacks, while others may strictly require complete relief of symptoms. Thus, individualized treatment is recommended, and adequate communication regarding the prognosis of the disease, the adverse effects of the medication and the expected outcome of the treatment is warranted before the use of medication. The initial dosage of carbamazepine for PKD treatment is recommended to be 50 mg and can be adjusted according to the practical effect [9, 66]. Regarding oxcarbazepine, 75 mg is recommended initially [9]. As the weight varies in children, the initial dosage of carbamazepine for pediatric patients with PKD can be set to 1 mg/kg and gradually titrated to the appropriate dosage. Furthermore, although most patients can well tolerate carbamazepine, the dizziness caused by carbamazepine may disturb the patients’ daily activities. Thus the medication should be taken at bedtime to minimize this adverse effect. In addition, as carbamazepine may result in Steven-Johnson syndrome/toxic epidermal necrolysis particularly in the Han Chinese population, HLA-B*15:02 screening should be implemented before initiating treatment to reduce the risk of adverse cutaneous reactions [9, 12]. For patients who harbor HLA-B*15:02 or cannot tolerate the dizziness or drowsiness of carbamazepine, other voltage-gated sodium channel blockers, including lamotrigine, topiramate, and phenytoin sodium, are recommended as the second-line treatment [9].

### Psychotherapy

Mental stress can increase the frequency and severity of PKD attacks. In addition, research on the psychological status of PKD patients revealed that PKD attacks can cause a certain degree of negative effect on patients, particularly adolescent patients. About one third of PKD patients have varying degrees of anxiety and depression [54]. Therefore, it is important to avoid stress, sleep deprivation, anxiety, and other psychological triggers that may increase the likelihood of PKD episodes, in order to prevent attacks and/or reduce the attack frequency. The psychological stress of patients is mainly attributed to the lack of knowledge about the etiology, development and prognosis of the disease. Thus, education among patients is particularly necessary to help them understand that PKD is a benign disease with a tendency toward natural remission to eliminate the psychological effects on patients’ lives and work.

### Special populations

#### a. Patients with BFIE

BFIE is an infantile cluster epilepsy that generally has a complete recovery [67]. Most BFIE cases are caused by *PRRT2* mutations, while mutations in other genes, including *SCN2A, SCN8A,* and *KCNQ2,* can also contribute. Therefore, genetic screening can assist in subsequent management. However, no specific interventions have been known to decrease the risk of subsequent development of PKD in BFIE patients, even in those with *PRRT2* mutations [7]. For BFIE patients carrying *PRRT2* mutations, carbamazepine or oxcarbazepine tends to be the preferred anti-epileptic drug (AED) because of the known favorable response in patients with PKD, although the responses to these drugs have not been well studied in patients with BFIE [7]. Benzodiazepines, including lorazepam, diazepam and midazolam, can be used to treat seizures lasting longer than 5 min or seizure clusters [7]. However, the response of *PRRT2*-associated seizures to benzodiazepines is less robust [7].

#### b. Pregnancy management

Some female patients have reported alleviation of attack frequency during pregnancy, but the underlying mechanism is unclear. However, prenatal exposure to AEDs may increase the risk of adverse fetal outcomes (depending on the type and dose of the drug and the stage of pregnancy at which medication is taken). Ideally, a comprehensive evaluation of the risks and benefits of AED medication during pregnancy should be conducted prior to conception [7]. For female patients with mild manifestations of PKD, discontinuing AED therapy prior to or during pregnancy should be considered due to the fetal risk related to AED therapy [7].

## Conclusions

PKD is a type of paroxysmal dyskinesia with high clinical and genetic heterogeneity. Although PKD is the most common type of paroxysmal dyskinesia, its low prevalence makes it a rare condition. This expert consensus on PKD diagnosis and treatment was proposed based on several large-scale clinical and genetic analyses of patients with PKD in domestic cohorts, in order to help establish standardized clinical evaluations and therapies for PKD. The diagnosis of PKD is based mainly on clinical features, and necessary evaluations are needed to exclude secondary etiologies. Personalized medical therapy and psychotherapy are recommended.

## Data Availability

Not applicable.

## Abbreviations

PKD: paroxysmal kinesigenic dyskinesia
*PRRT2*: proline-rich transmembrane protein 2
PNKD: paroxysmal nonkinesigenic dyskinesia
PED: paroxysmal exercise-induced dyskinesia
BFIE: benign familial infantile epilepsy
AED: anti-epileptic drug

## References

[1] Kertesz A (1967). Paroxysmal kinesigenic choreoathetosis. An entity within the paroxysmal choreoathetosis syndrome. Description of 10 cases, including 1 autopsied. Neurology.

[2] Bruno MK, Hallett M, Gwinn-Hardy K, Sorensen B, Considine E, Tucker S (2004). Clinical evaluation of idiopathic paroxysmal kinesigenic dyskinesia: new diagnostic criteria. Neurology.

[3] Tomita H, Nagamitsu S, Wakui K, Fukushima Y, Yamada K, Sadamatsu M (1999). Paroxysmal kinesigenic choreoathetosis locus maps to chromosome 16p11.2-q12.1. Am J Hum Genet.

[4] Chen WJ, Lin Y, Xiong ZQ, Wei W, Ni W, Tan GH (2011). Exome sequencing identifies truncating mutations in *PRRT2* that cause paroxysmal kinesigenic dyskinesia. Nat Genet.

[5] Wang JL, Cao L, Li XH, Hu ZM, Li JD, Zhang JG (2011). Identification of *PRRT2* as the causative gene of paroxysmal kinesigenic dyskinesias. Brain.

[6] Bhatia KP, Schneider SA (2012). Identification of *PRRT2* as the causative gene of paroxysmal kinesigenic dyskinesia. Mov Disord.

[7] Ebrahimi-Fakhari D, Moufawad El Achkar C, Klein C, Adam MP, Ardinger HH, Pagon RA (1993). *PRRT2*-associated paroxysmal movement disorders. GeneReviews.

[8] Ebrahimi-Fakhari D, Saffari A, Westenberger A, Klein C (2015). The evolving spectrum of PRRT2-associated paroxysmal diseases. Brain.

[9] Huang XJ, Wang SG, Guo XN, Tian WT, Zhan FX, Zhu ZY (2020). The phenotypic and genetic spectrum of paroxysmal kinesigenic dyskinesia in China. Mov Disord.

[10] Bhatia KP (2011). Paroxysmal dyskinesias. Mov Disord.

[11] Kim SY, Lee JS, Kim WJ, Kim H, Choi SA, Lim BC (2018). Paroxysmal dyskinesia in children: from genes to the clinic. J Clin Neurol.

[12] Huang XJ, Wang T, Wang JL, Liu XL, Che XQ, Li J (2015). Paroxysmal kinesigenic dyskinesia: clinical and genetic analyses of 110 patients. Neurology.

[13] Latorre A, Bhatia KP (2020). Treatment of paroxysmal dyskinesia. Neurol Clin.

[14] Tan LC, Methawasin K, Teng EW, Ng AR, Seah SH, Au WL (2014). Clinico-genetic comparisons of paroxysmal kinesigenic dyskinesia patients with and without *PRRT2* mutations. Eur J Neurol.

[15] Erro R, Sheerin UM, Bhatia KP (2014). Paroxysmal dyskinesias revisited: a review of 500 genetically proven cases and a new classification. Mov Disord.

[16] Cloarec R, Bruneau N, Rudolf G, Massacrier A, Salmi M, Bataillard M (2012). *PRRT2* links infantile convulsions and paroxysmal dyskinesia with migraine. Neurology.

[17] Erro R, Bhatia KP (2019). Unravelling of the paroxysmal dyskinesias. J Neurol Neurosurg Psychiatry.

[18] Delcourt M, Riant F, Mancini J, Milh M, Navarro V, Roze E (2015). Severe phenotypic spectrum of biallelic mutations in *PRRT2* gene. J Neurol Neurosurg Psychiatry.

[19] Weber A, Kohler A, Hahn A, Neubauer B, Muller U (2013). Benign infantile convulsions (IC) and subsequent paroxysmal kinesigenic dyskinesia (PKD) in a patient with 16p11.2 microdeletion syndrome. Neurogenetics.

[20] Demirkiran M, Jankovic J (1995). Paroxysmal dyskinesias: clinical features and classification. Ann Neurol.

[21] van Vliet R, Breedveld G, de Rijk-van Andel J, Brilstra E, Verbeek N, Verschuuren-Bemelmans C (2012). *PRRT2* phenotypes and penetrance of paroxysmal kinesigenic dyskinesia and infantile convulsions. Neurology.

[22] Yin XM, Lin JH, Cao L, Zhang TM, Zeng S, Zhang KL (2018). Familial paroxysmal kinesigenic dyskinesia is associated with mutations in the *KCNA1* gene. Hum Mol Genet.

[23] Tian WT, Huang XJ, Mao X, Liu Q, Liu XL, Zeng S (2018). Proline-rich transmembrane protein 2-negative paroxysmal kinesigenic dyskinesia: clinical and genetic analyses of 163 patients. Mov Disord.

[24] Gardella E, Becker F, Moller RS, Schubert J, Lemke JR, Larsen LH (2016). Benign infantile seizures and paroxysmal dyskinesia caused by an *SCN8A* mutation. Ann Neurol.

[25] Gardiner AR, Jaffer F, Dale RC, Labrum R, Erro R, Meyer E (2015). The clinical and genetic heterogeneity of paroxysmal dyskinesias. Brain.

[26] Wang HX, Li HF, Liu GL, Wen XD, Wu ZY (2016). Mutation analysis of *MR-1*, *SLC2A1*, and *CLCN1* in 28 PRRT2-negative paroxysmal kinesigenic dyskinesia patients. Chin Med J.

[27] Fabbri M, Marini C, Bisulli F, Di Vito L, Elia A, Guerrini R (2013). Clinical and polygraphic study of familial paroxysmal kinesigenic dyskinesia with *PRRT2* mutation. Epileptic Disord.

[28] Groffen AJ, Klapwijk T, van Rootselaar AF, Groen JL, Tijssen MA (2013). Genetic and phenotypic heterogeneity in sporadic and familial forms of paroxysmal dyskinesia. J Neurol.

[29] Cao L, Huang XJ, Zheng L, Xiao Q, Wang XJ, Chen SD (2012). Identification of a novel *PRRT2* mutation in patients with paroxysmal kinesigenic dyskinesias and c.649dupC as a mutation hot-spot. Parkinsonism Relat Disord.

[30] Valente P, Castroflorio E, Rossi P, Fadda M, Sterlini B, Cervigni RI (2016). PRRT2 is a key component of the Ca (2+)-dependent neurotransmitter release machinery. Cell Rep.

[31] Liu YT, Nian FS, Chou WJ, Tai CY, Kwan SY, Chen C (2016). *PRRT2* mutations lead to neuronal dysfunction and neurodevelopmental defects. Oncotarget.

[32] Fruscione F, Valente P, Sterlini B, Romei A, Baldassari S, Fadda M (2018). PRRT2 controls neuronal excitability by negatively modulating Na+ channel 1.2/1.6 activity. Brain.

[33] Joo EY, Hong SB, Tae WS, Kim JH, Han SJ, Seo DW (2005). Perfusion abnormality of the caudate nucleus in patients with paroxysmal kinesigenic choreoathetosis. Eur J Nucl Med Mol Imaging.

[34] Shirane S, Sasaki M, Kogure D, Matsuda H, Hashimoto T (2001). Increased ictal perfusion of the thalamus in paroxysmal kinesigenic dyskinesia. J Neurol Neurosurg Psychiatry.

[35] Zhou B, Chen Q, Zhang Q, Chen L, Gong Q, Shang H (2010). Hyperactive putamen in patients with paroxysmal kinesigenic choreoathetosis: a resting-state functional magnetic resonance imaging study. Mov Disord.

[36] Kim MO, Im JH, Choi CG, Lee MC (1998). Proton MR spectroscopic findings in paroxysmal kinesigenic dyskinesia. Mov Disord.

[37] Li HF, Yang L, Yin D, Chen WJ, Liu GL, Ni W (2019). Associations between neuroanatomical abnormality and motor symptoms in paroxysmal kinesigenic dyskinesia. Parkinsonism Relat Disord.

[38] Long Z, Xu Q, Miao HH, Yu Y, Ding MP, Chen H (2017). Thalamocortical dysconnectivity in paroxysmal kinesigenic dyskinesia: combining functional magnetic resonance imaging and diffusion tensor imaging. Mov Disord.

[39] Blakeley J, Jankovic J (2002). Secondary paroxysmal dyskinesias. Mov Disord.

[40] Berger JR, Sheremata WA, Melamed E (1984). Paroxysmal dystonia as the initial manifestation of multiple sclerosis. Arch Neurol.

[41] Cottrill N, McCully B, Payne M (2019). Paroxysmal kinesigenic dyskinesia presented following concussion. J Mov Disord.

[42] Candeias da Silva C, Bichuetti DB, Azevedo Silva SMC, Ferraz HB, Oliveira EML, Borges V (2018). Movement disorders in multiple sclerosis and neuromyelitis optica: a clinical marker of neurological disability. Parkinsonism Relat Disord.

[43] Frohlich K, Winder K, Linker RA, Huhn K, Engelhorn T, Dorfler A (2018). Lesion correlates of secondary paroxysmal dyskinesia in multiple sclerosis. J Neurol.

[44] Ciampi E, Uribe-San-Martin R, Godoy-Santin J, Cruz JP, Carcamo-Rodriguez C, Juri C (2017). Secondary paroxysmal dyskinesia in multiple sclerosis: clinical-radiological features and treatment. Case report of seven patients. Mult Scler.

[45] Pop R, Kipfer S (2017). Paroxysmal kinesigenic dyskinesia-like phenotype in multiple sclerosis. Mult Scler.

[46] Baguma M, Ossemann M (2017). Paroxysmal kinesigenic dyskinesia as the presenting and only manifestation of multiple sclerosis after eighteen months of follow-up. J Mov Disord.

[47] Kostic VS, Petrovic IN (2017). Brain calcification and movement disorders. Curr Neurol Neurosci Rep.

[48] Wang C, Ma X, Xu X, Huang B, Sun H, Li L (2017). A *PDGFB* mutation causes paroxysmal nonkinesigenic dyskinesia with brain calcification. Mov Disord.

[49] Kwon YJ, Jung JM, Choi JY, Kwon DY (2015). Paroxysmal kinesigenic dyskinesia in pseudohypoparathyroidism: is basal ganglia calcification a necessary finding?. J Neurol Sci.

[50] Zhu M, Zhu X, Wan H, Hong D (2014). Familial IBGC caused by *SLC20A2* mutation presenting as paroxysmal kinesigenic dyskinesia. Parkinsonism Relat Disord.

[51] Chung EJ, Cho GN, Kim SJ (2012). A case of paroxysmal kinesigenic dyskinesia in idiopathic bilateral striopallidodentate calcinosis. Seizure.

[52] Silveira-Moriyama L, Kovac S, Kurian MA, Houlden H, Lees AJ, Walker MC (2018). Phenotypes, genotypes, and the management of paroxysmal movement disorders. Dev Med Child Neurol.

[53] Lance JW (1977). Familial paroxysmal dystonic choreoathetosis and its differentiation from related syndromes. Ann Neurol.

[54] Tian WT, Huang XJ, Liu XL, Shen JY, Liang GL, Zhu CX (2017). Depression, anxiety, and quality of life in paroxysmal kinesigenic dyskinesia patients. Chin Med J.

[55] Waln O, Jankovic J (2015). Paroxysmal movement disorders. Neurol Clin.

[56] Ganos C, Aguirregomozcorta M, Batla A, Stamelou M, Schwingenschuh P, Munchau A (2014). Psychogenic paroxysmal movement disorders--clinical features and diagnostic clues. Parkinsonism Relat Disord.

[57] Bakker MJ, van Dijk JG, van den Maagdenberg AM, Tijssen MA (2006). Startle syndromes. Lancet Neurol.

[58] Thomas RH, Chung SK, Wood SE, Cushion TD, Drew CJ, Hammond CL (2013). Genotype-phenotype correlations in hyperekplexia: apnoeas, learning difficulties and speech delay. Brain.

[59] Gherpelli JL, Nogueira AR, Troster EJ, Deutsch AD, Leone CR, Brotto MW (1995). Hyperekplexia, a cause of neonatal apnea: a case report. Brain and Development.

[60] Koning-Tijssen MA, Brouwer OF (2000). Hyperekplexia in the first year of life. Mov Disord.

[61] Kirkham FJ, Haywood P, Kashyape P, Borbone J, Lording A, Pryde K (2011). Movement disorder emergencies in childhood. Eur J Paediatr Neurol.

[62] Garone G, Capuano A, Travaglini L, Graziola F, Stregapede F, Zanni G, et al. Clinical and genetic overview of paroxysmal movement disorders and episodic ataxias. Int J Mol Sci. 2020;21(10):3603. 10.3390/ijms21103603 PMC7279391 32443735

[63] Canavese C, Canafoglia L, Costa C, Zibordi F, Zorzi G, Binelli S (2012). Paroxysmal non-epileptic motor events in childhood: a clinical and video-EEG-polymyographic study. Dev Med Child Neurol.

[64] Bonnet C, Roubertie A, Doummar D, Bahi-Buisson N, Cochen de Cock V, Roze E (2010). Developmental and benign movement disorders in childhood. Mov Disord.

[65] Yang Y, Su Y, Guo Y, Ding Y, Xu S, Jiang Y (2012). Oxcarbazepine versus carbamazepine in the treatment of paroxysmal kinesigenic dyskinesia. Int J Neurosci.

[66] Li HF, Chen WJ, Ni W, Wang KY, Liu GL, Wang N (2013). *PRRT2* mutation correlated with phenotype of paroxysmal kinesigenic dyskinesia and drug response. Neurology.

[67] van Roest A, Van de Vel A, Lederer D, Ceulemans B (2020). The clinical and genetic spectrum in infants with (an) unprovoked cluster(s) of focal seizures. Eur J Paediatr Neurol.

